# Nutritional Relationship between *Bemisia tabaci* and Its Primary Endosymbiont, *Portiera aleyrodidarum*, during Host Plant Acclimation

**DOI:** 10.3390/insects11080498

**Published:** 2020-08-04

**Authors:** Fang-Yu Hu, Chi-Wei Tsai

**Affiliations:** Department of Entomology, National Taiwan University, Taipei 106, Taiwan; r06632002@ntu.edu.tw

**Keywords:** cotton, developmental time, essential amino acids, fecundity, survival rate

## Abstract

**Simple Summary:**

Plant sap-sucking insects commonly have established mutualistic relationships with bacteria that live within their bodies and often provide nutrients that are lacking in the insect’s diet. The sweet potato whitefly (*Bemisia tabaci*) harbors one primary and up to seven secondary endosymbiotic bacteria. The primary endosymbiont of *B. tabaci* is already known to play a critical role in providing necessary nutrients for *B. tabaci*. Our objective was to study the relationship among *B. tabaci*, its primary endosymbiont, and the host plant through the effects of host plant shifting and acclimation, that is, physiological adjustments as an insect becomes accustomed to a new host plant over several generations. The results showed that host shifting from Chinese kale to cotton plants led to a decrease in the fecundity of *B. tabaci* in the first generation, which was restored after 10 generations of acclimation, and that its developmental time was also decreased by the tenth generation. Furthermore, essential amino acid biosynthesis genes of its primary endosymbiont were differentially regulated after *B. tabaci* had become acclimated to cotton plants. We speculate that the primary endosymbiont has a close nutritional relationship with *B. tabaci* during host plant acclimation.

**Abstract:**

Plant sap-sucking insects commonly have established mutualistic relationships with endosymbiotic bacteria that can provide nutrients lacking in their diet. *Bemisia tabaci* harbors one primary endosymbiont, *Portiera aleyrodidarum*, and up to seven secondary endosymbionts, including *Hamiltonella defensa* and *Rickettsia* sp. *Portiera aleyrodidarum* is already known to play a critical role in providing necessary nutrients for *B. tabaci*. In the present study, the relationship among *B. tabaci*, its primary endosymbiont, and the host plant were examined through the effects of host plant shifting and acclimation. *Bemisia tabaci* was transferred from Chinese kale to four different host plants, and the effects on both its performance and the expression levels of nutrient-related genes of *P. aleyrodidarum* were analyzed. The results showed that host shifting from Chinese kale to cotton plants led to a decrease in the performance of *B. tabaci* in the first generation, which was restored after 10 generations of acclimation. Furthermore, the expression levels of essential amino acid biosynthesis genes of *P. aleyrodidarum* were found to be differentially regulated after *B. tabaci* had acclimated to the cotton plants. Host plant shifting and acclimation to cucumber, poinsettia, and tomato plants did not affect the fecundity of *B. tabaci* and the expression levels of most examined genes. We speculate that *P. aleyrodidarum* may help *B. tabaci* improve its performance and acclimate to new hosts and that *P. aleyrodidarum* has a close nutritional relationship with its host during host plant acclimation.

## 1. Introduction

Hemipteran insects commonly harbor endosymbiotic bacteria [1], which can be categorized into two groups. Primary endosymbionts, which can synthesize necessary nutrients lacked by their hosts, are often located in specialized cells called bacteriocytes and have an obligate mutualistic relationship with their host [2]. Secondary endosymbionts may occur in bacteriocytes or in other tissues and usually have a facultative symbiotic relationship with their host. While primary endosymbionts are normally vertically transmitted from mother to offspring, secondary endosymbionts can be transmitted either vertically or horizontally [3].

Endosymbionts in insects have diverse functions, such as defense against natural enemies [4,5], defense against pathogens [6,7], body color [8], thermal tolerance [9,10], susceptibility to insecticides [11,12], vector competence to plant viruses [13,14,15], and reproduction [16]. They can be mutualistic, neutral, or parasitic to host insects [17]. Generally, these effects are associated with secondary endosymbionts.

*Bemisia tabaci* (Hemiptera: Aleyrodidae) is a species complex including at least 39 morphologically indistinguishable species [18,19,20], of which Middle East-Asia Minor 1 (MEAM1) putative species and Mediterranean (MED) putative species are widely distributed throughout the world. To date, *B. tabaci* species complex has been reported to harbor one primary endosymbiont, *Portiera aleyrodidarum*, and up to seven secondary endosymbionts, *Hamiltonella defensa*, *Rickettsia* sp., *Wolbachia* sp., *Arsenophonus* sp., *Fritschea* sp., *Cardinium* sp., and *Hemipteriohilus* sp. [21].

*Bemisia tabaci* is a polyphagous insect that can survive and reproduce on various host plants. To survive on a new host plant, *B. tabaci* needs physiological adjustment to be accustomed to the distinct physiological condition of the new host, which is termed acclimation. Malka et al. [22] suggests that *B. tabaci* MEAM1 has a detoxification mechanism, which helps it acclimate to new host plants. Besides the detoxification capability of *B. tabaci* itself, the primary endosymbiont, *P. aleyrodidarum*, of *B. tabaci* also plays a critical role in providing necessary nutrients for its hosts. An analysis of the *P. aleyrodidarum* genome has identified numerous biosynthesis genes of essential amino acids and carotenoids that are lacking in the genome and the diet of *B. tabaci*, indicating that *P. aleyrodidarum* has a nutritional mutualistic relationship with *B. tabaci* [23,24,25,26,27]. *Buchnera* sp. in *Acyrthosiphon pisum* has also been shown to possess crucial genes for amino acid biosynthesis and cooperates with its host to produce essential amino acids [28]. Santos-Garcia et al. [29] also reported that the gut-associated bacteria in *B. tabaci* MEAM1 may be beneficial during host plant shifting and acclimation. 

When a polyphagous insect feeds and lives on a new host plant, it becomes accustomed to the distinct nutritional condition of the new host by adjusting its physiology. Due to the nutritional mutualistic relationship between *P. aleyrodidarum* and *B. tabaci*, we hypothesized that host plant acclimation is associated with this nutritional mutualistic endosymbiont. To advance the current knowledge of endosymbionts in plant sap-sucking insects, the present study determined the effects of host plant shifting and acclimation on the performance of *B. tabaci* MEAM1. Furthermore, the expression of nutrient-related genes of *P. aleyrodidarum* were examined after host plant shifting and acclimation. Unfortunately, the genes of *P. aleyrodidarum* are still unable to be manipulated artificially to date, including knockdown and/or overexpression, so we cannot directly demonstrate whether *P. aleyrodidarum* helps *B. tabaci* improve its performance and acclimate to new host plants. This is the first study examining changes in the expression of nutrition-related genes of insect endosymbiotic bacteria in relation to host plant acclimation.

## 2. Materials and Methods

### 2.1. Insect and Plants

A colony of *B. tabaci* MEAM1 species (mtCO1 sequence is identical to GenBank accession number EU427726) [30] was reared on Chinese kale (*Brassica oleracea* cv. Alboglabra Group) for five years (more than 85 generations) in insect-proof net cages (30 × 30 × 30 cm, Megaview, Taichung, Taiwan) at 28 ± 2 °C and under a photoperiod of L:D 16:8 h. The laboratory colony was confirmed as harboring *P. aleyrodidarum*, *H. defensa*, and *Rickettsia* sp. by a PCR assay as described in Ansari et al. [31], and they were free of *Wolbachia* sp., *Arsenophonus* sp., *Fritschea* sp., *Cardinium* sp., and *Hemipteriohilus* sp.

The host plants of *B. tabaci* used for the present study were Chinese kale, cotton (*Gossypium barbadense*), cucumber (*Cucumis sativus* cv. hualu), poinsettia (*Euphorbia pulcherrima*), and tomato (*Solanum lycopersicum* cv. ANT22), which are common host plants of *B. tabaci* MEAM1 from different families. Chinese kale, cotton, cucumber and tomato plants were grown from seeds, and poinsettia plants were propagated by cutting. All plants were grown at 28 ± 2 °C and under a photoperiod of L:D 16:8 h in environmental chambers. For the experiments, Chinese kale, cotton and tomato plants with 4–6 leaves, cucumber plants with 3–4 leaves, and poinsettia with 10–15 leaves were used.

### 2.2. Host Plant Shifting and Acclimation

Groups of 100 whitefly individuals (sex ratio 1:1) from the original colony reared on Chinese kale were transferred to insect-proof net cages (22 × 22 × 22 cm, Megaview). A total of five cages were used, each containing two plants of either Chinese kale, cotton, cucumber, poinsettia, or tomato. Two weeks later, all adult whiteflies were removed from the plants using a mouth aspirator, such that eggs and nymphs remained on the leaves. After the nymphs emerged to adults, 100 of the newly-emerged adult whiteflies were transferred to new net cages, each enclosed with two new host plants of the same species to breed the next generation. The whitefly colonies were reared on each host plant for 10 continuous generations.

### 2.3. Performance of B. tabaci

To determine the effects of host plant shifting and acclimation on the performance of *B. tabaci*, adult females were collected from the first and tenth generations on each host plant. Adult females were enclosed individually with a leaf of each host plant in a gauze bag (6 × 15 cm, 109 mesh, Megaview) for oviposition. After 72 h, the female was removed and the number of eggs counted using a stereomicroscope to determine its fecundity. The number of newly-emerged adults developing from the eggs was also recorded daily to calculate the developmental time and the survival rate of “egg and nymphal stages”. The performance assay was conducted with 20 replicates per host plant for original, G1, and G10 colonies. The original colony represented the colony reared on Chinese kale. The data of original and G1 colonies were compared to examine the effect of host plant shifting, and the data of G1 and G10 colonies were compared to examine the effect of acclimation.

### 2.4. Expression of Nutrient-Related Genes of P. aleyrodidarum

To interpret the relationship between host plant shifting and acclimation and the expression of *P. aleyrodidarum* nutrient-related genes, the expression levels of essential amino acid biosynthesis genes (*trpA*, *trpB*, *leuB*, *leuC*, *ilvC*, and *ilvD*), which are encoded only in the genome of *P*. *aleyrodidarum* [26], were analyzed via a reverse transcription quantitative polymerase chain reaction (RT-qPCR) assay. Tryptophan, leucine and isoleucine biosynthesis cannot be completed by *B. tabaci* itself, and most essential genes in biosynthesis pathways are only present in *P. aleyrodidarum*. Therefore, we chose to examine these six genes from three amino acid biosynthesis pathways. For both the first and tenth generations of whiteflies, 25 males and 25 females were collected from each host plant. Each set of 5 males or 5 females was pooled to make single samples. Males and females were analyzed separately because females need more nutrients than males for reproduction [32]. The samples were then homogenized with 10 µL of RLT buffer in a 1.5-mL eppendorf tube using a micropestle. Total RNA was extracted using a RNeasy Mini Kit (Qiagen, Valencia, CA, USA) following the manufacturer’s instructions. Extracted RNA was then treated with a TURBO DNA-free Kit (Invitrogen, Carlsbad, CA, USA) to eliminate residual DNA. cDNA was synthesized from 500 ng of RNA using a PrimeScript RT Reagent Kit (Takara Bio Inc., Kusatsu, Shiga, Japan) according to the manufacturer’s instructions. SYBR green real-time PCR was then performed in a 20-µL reaction volume, containing 10 µL of 2 × SensiFAST SYBR Hi-ROX Mix (Bioline, London, UK), 7 µL of ddH_2_O, 0.5 µL of each primer (10 µM), and 2 µL of the cDNA template. The primer sets used were gene-specific (Table 1) and designed in the present study by Primer Express Software (Applied Biosystems, Foster City, CA, USA) using the default criteria. The cycling conditions were as follows: one cycle of denaturation (95 °C, 5 min), followed by 40 cycles of denaturation (95 °C, 5 s), annealing (60 °C, 10 s), and extension (72 °C, 20 s). The PCR efficiency of *trpA*, *trpB*, *leuB*, *leuC*, *ilvC*, *ilvD*, and *gyrA* specific primer sets were 98.9%, 104.0%, 104.0%. 100.4%, 98.5%, 102.7%, and 105.6%, respectively. Melting curves were generated at the end of the reaction to confirm the specificity of the amplification.

The relative expression levels of the essential amino acid biosynthesis genes (*trpA*, *trpB*, *leuB*, *leuC*, *ilvC*, and *ilvD*) of *P. aleyrodidarum* were calculated using the 2^−ΔCt^ method. *gyrA* of *P. aleyrodidarum* was used as a normalization gene [33]. Each experiment was conducted using pooled samples of whiteflies from each host plant in the first and tenth generations with five biological replicates.

### 2.5. Statistical Analyses

The performance data of *B. tabaci* were analyzed by Kruskal-Wallis test with post-hoc Dunn’s test. The gene expression data were first performed with normality transformation (logarithm transformation) and were then analyzed by one-way analysis of variance (ANOVA). When significant differences were observed, the Tukey test was employed as a post-hoc test after ANOVA. All data analyses were performed using SAS 9.4 (SAS Institute Inc., Cary, NC, USA).

## 3. Results

### 3.1. Effects of Host Plant Shifting and Acclimation on the Performance of B. tabaci

**Figure 1:**
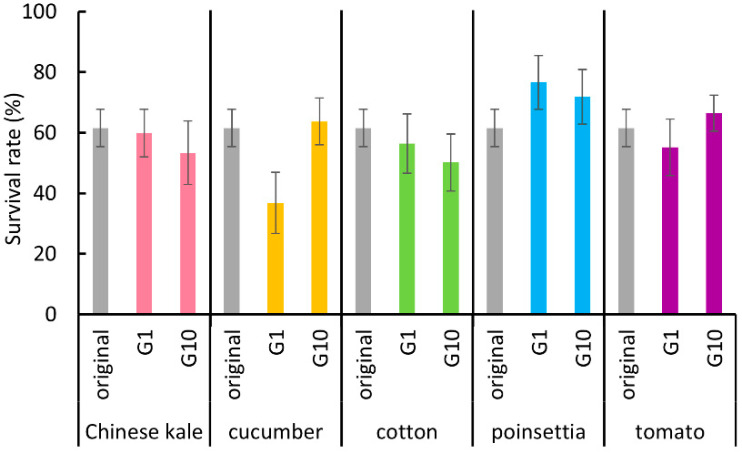
The survival rate of “egg and nymphal stages” of *B. tabaci* reared on each host plant in the first and tenth generations, and the original colony. Vertical bars indicate standard errors. There were no significant differences (Kruskal-Wallis test, *p* > 0.05).

After the whiteflies had been reared on new host plants for 10 generations, they were considered acclimated to the new host plant. Thus, their performance at the first and tenth generations was evaluated and compared with that of the original colony. The survival rates of “egg and nymphal stages” of the first-generation whiteflies reared on each new host plant were not significantly different to those of the whiteflies from the original colony (Figure 1, Kruskal–Wallis test, Chinese kale: *p* = 0.52; cucumber: *p* = 0.10; cotton: *p* = 0.80; poinsettia: *p* = 0.10; tomato: *p* = 0.76). The survival rates were also not significantly different after the whiteflies had become acclimated to each new host plant for 10 generations (Figure 1, Kruskal–Wallis test, Chinese kale: *p* = 0.52; cucumber: *p* = 0.10; cotton: *p* = 0.80; poinsettia: *p* = 0.10; tomato: *p* = 0.76).

**Figure 2:**
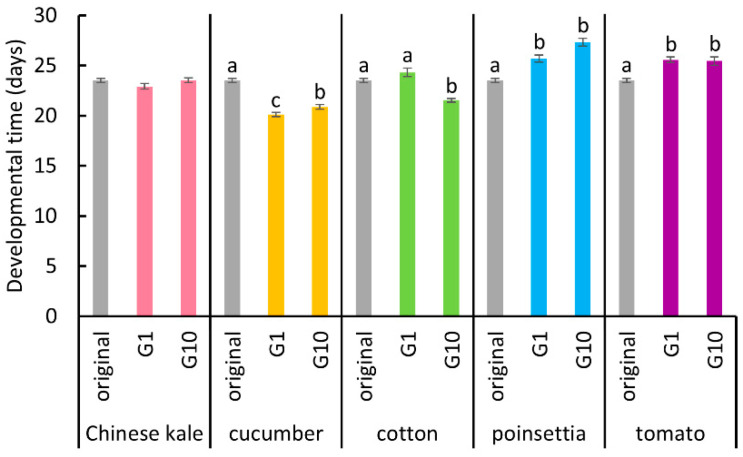
The developmental time of “egg and nymphal stages” of *B. tabaci* reared on each host plant in the first and tenth generations, and the original colony. Vertical bars indicate standard errors. Different letters indicate significant differences (Dunn’s test, *p* < 0.05).

Regarding developmental time of “egg and nymphal stages”, the transfer from Chinese kale to cucumber plants resulted in a significant decrease for the first generation, which slightly lengthened by the tenth generation (Figure 2, Dunn’s test, *p* < 0.05). For the group transferred from Chinese kale to cotton plants, developmental time for the first generation was not significantly different to that of the original colony (Dunn’s test, *p* > 0.05) but was significantly decreased by the tenth generation (Figure 2, Dunn’s test, *p* < 0.05). For the groups transferred from Chinese kale to poinsettia and tomato plants, respectively, developmental time was significantly longer in comparison to the original colony for both generations (Figure 2, Dunn’s test, *p* < 0.05).

**Figure 3:**
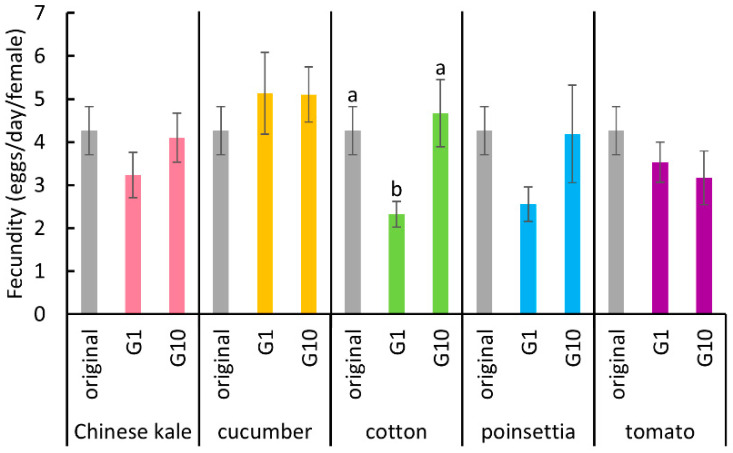
The fecundity of *B. tabaci* reared on each host plant in the first and tenth generations, and the original colony. Vertical bars indicate standard errors. Different letters indicate significant differences (Dunn’s test, *p* < 0.05).

The fecundity of the whiteflies was significantly reduced after they had been transferred from Chinese kale to cotton plants (Dunn’s test, *p* < 0.05) and then reverted to its original level after they had fed on cotton plants for 10 generations (Figure 3, Dunn’s test, *p* < 0.05). However, for transfers from Chinese kale to the other three host plants, no significant effects were observed (Figure 3, Kruskal-Wallis test, Chinese kale: *p* = 0.34; cucumber: *p* = 0.70; poinsettia: *p* = 0.08; tomato: *p* = 0.37).

Taken together, it was found that host plant shifting and acclimation affect the performance of *B. tabaci*, especially on cotton plants.

### 3.2. Relationship between Host Plant Shifting and Acclimation and the Expression of Nutrient-Related Genes of P. aleyrodidarum

**Figure 4:**
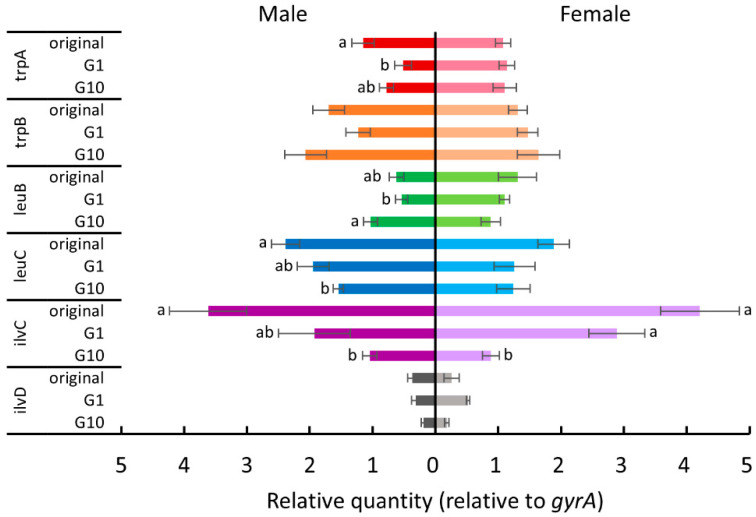
The expression levels of tryptophan, leucine, and isoleucine biosynthesis genes of *P. aleyrodidarum* in male and female *B. tabaci* reared on cucumber plants in the first and the tenth generations. Expression levels in the original colony are also shown. Horizontal bars indicate standard errors. Different letters indicate significant differences (Tukey test, *p* < 0.05).

**Figure 5:**
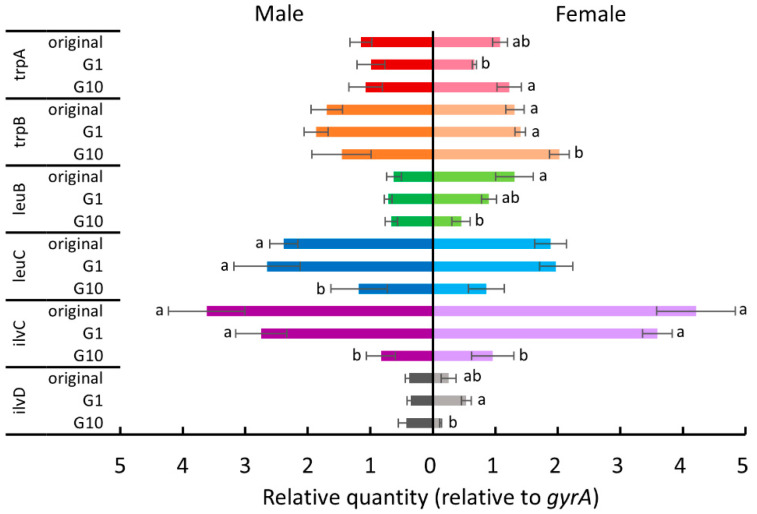
The expression levels of tryptophan, leucine, and isoleucine biosynthesis genes of *P. aleyrodidarum* in male and female *B. tabaci* reared on cotton plants in the first and the tenth generations. Expression levels in the original colony are also shown. Horizontal bars indicate standard errors. Different letters indicate significant differences (Tukey test, *p* < 0.05).

**Figure 6:**
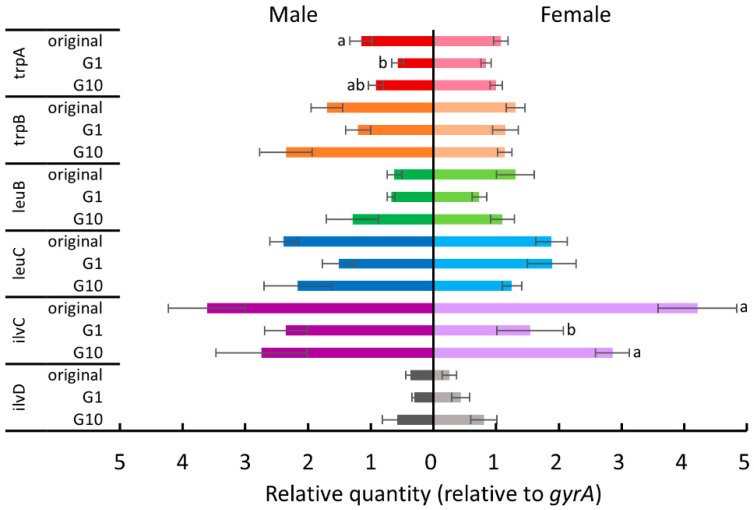
The expression levels of tryptophan, leucine, and isoleucine biosynthesis genes of *P. aleyrodidarum* in male and female *B. tabaci* reared on poinsettia in the first and the tenth generations. Expression levels in the original colony are also shown. Horizontal bars indicate standard errors. Different letters indicate significant differences (Tukey test, *p* < 0.05).

**Figure 7:**
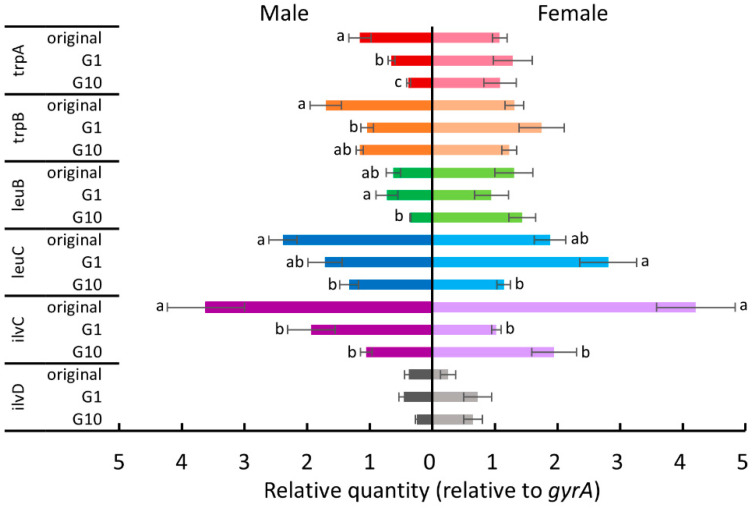
The expression levels of tryptophan, leucine, and isoleucine biosynthesis genes of *P. aleyrodidarum* in male and female *B. tabaci* reared on tomato plants in the first and the tenth generations. Expression levels in the original colony are also shown. Horizontal bars indicate standard errors. Different letters indicate significant differences (Tukey test, *p* < 0.05).

Since *B. tabaci* depends on *P. aleyrodidarum* to supplement several essential amino acids [27], we hypothesized that host plant acclimation is associated with this nutritional mutualistic endosymbiont. Therefore, we examined the expression levels of some tryptophan, leucine, and isoleucine biosynthesis genes (*trpA*, *trpB*, *leuB*, *leuC*, *ilvC*, and *ilvD*) of *P. aleyrodidarum* for the whiteflies reared on each new host plant in the first and the tenth generations. Figure 4, Figure 5, Figure 6 and Figure 7 show the expression levels after *B. tabaci* was transferred to cucumber, cotton, poinsettia, and tomato, respectively.

For female whiteflies transferred to cucumber plants (Figure 4), *ilvC* expression was significantly downregulated in tenth-generation whiteflies (Tukey test, *p* < 0.05). For males transferred to cucumber plants (Figure 4), *trpA* expression was significantly downregulated in the first generation (Tukey test, *p* < 0.05), and *leuB* expression was significantly upregulated after 10 generations (Tukey test, *p* < 0.05). The expression of both *leuC* and *ilvC* in tenth-generation males was significantly downregulated compared to that in the original colony (Tukey test, *p* < 0.05).

For female whiteflies transferred to cotton plants (Figure 5), most genes were differentially expressed between the first and the tenth generations (Tukey test, *p* < 0.05). The expression of both *trpA* and *trpB* was significantly upregulated (Tukey test, *p* < 0.05), while that of *ilvC* and *ilvD* was significantly downregulated (Tukey test, *p* < 0.05). For males transferred to cotton plants (Figure 5), there were no significant differences in the expression of all examined genes (one way-ANOVA, *trpA*: *p* = 0.83; *trpB*: *p* = 0.37; *leuB*: *p* = 0.72; *ilvD*: *p* = 0.99) except for *leuC* and *ilvC*, which were downregulated after 10 generations (Tukey test, *p* < 0.05).

For female whiteflies transferred to poinsettia (Figure 6), *ilvC* expression was significantly downregulated in the first generation (Tukey test, *p* < 0.05) and upregulated after 10 generations (Tukey test, *p* < 0.05), whereas the expression of all other genes did not show significant differences between the first and tenth generations (one way-ANOVA, *trpA*: *p* = 0.23; *trpB*: *p* = 0.64; *leuB*: *p* = 0.20; *leuC*: *p* = 0.26; *ilvD*: *p* = 0.10). For males transferred to poinsettia (Figure 6), *trpA* expression was significantly downregulated in the first generation (Tukey test, *p* < 0.05). The other examined genes did not show differences in expression level after being transferred to this host plant (one way-ANOVA, *trpB*: *p* = 0.054; *leuB*: *p* = 0.71; *leuC*: *p* = 0.28; *ilvC*: *p* = 0.49; *ilvD*: *p* = 0.56).

For female whiteflies transferred to tomato plants (Figure 7), *ilvC* expression was significantly downregulated in the first generation (Tukey test, *p* < 0.05), and *leuC* expression was significantly downregulated after 10 generations (Tukey test, *p* < 0.05). For males transferred to tomato plants (Figure 7), the expression of *trpA*, *trpB*, and *ilvC* was significantly downregulated after being transferred to tomato plants (Tukey test, *p* < 0.05), and *trpA* expression was further downregulated after 10 generations (Tukey test, *p* < 0.05). In addition, *leuB* expression was significantly downregulated after 10 generations (Tukey test, *p* < 0.05), and *leuC* expression was significantly downregulated in the tenth generation compared to that of the original colony (Tukey test, *p* < 0.05).

**Figure 8:**
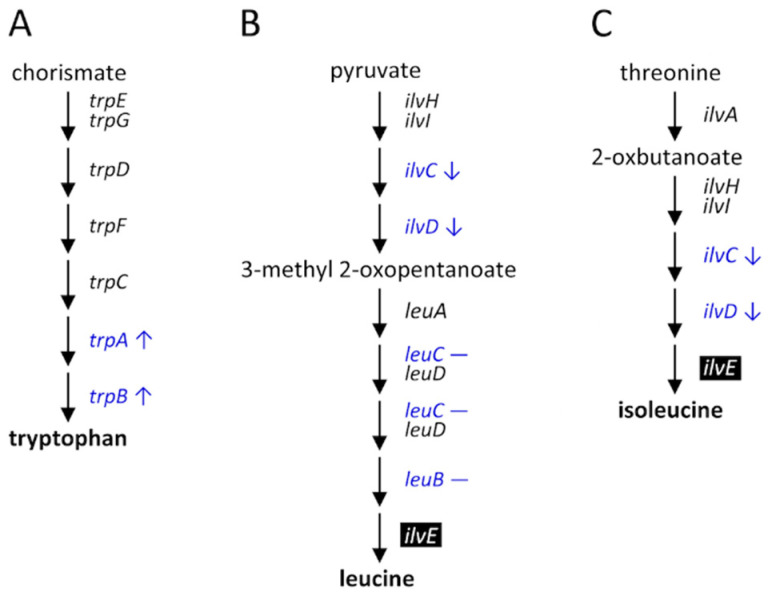
Differential expression of the genes in essential amino acid biosynthesis pathways of *P. aleyrodidarum* in female *B. tabaci* reared on cotton plants between the first and the tenth generations: (**A**) tryptophan, (**B**) leucine, and (**C**) isoleucine. Reverse type indicates genes that exist only in *B. tabaci* and blue font indicates those examined in the present study. Up and down arrows indicate that gene expression was up- and downregulated, respectively, in the tenth generation. Dash indicates no differential expression between the first and the tenth generations.

In summary, the biosynthesis of the essential amino acids provided by *P. aleyrodidarum* was regulated during host plant shifting and acclimation, especially when *B. tabaci* acclimated to cotton plants (Figure 8).

## 4. Discussion

In the present study, we investigated the effect of transferring *B. tabaci* from Chinese kale to four different host plants. Since the nutrient composition and defensive compounds of the phloem sap differ in each host plant, the performance of *B. tabaci* may be improved or impaired. This is reflected in our results, whereby the developmental time of *B. tabaci* was significantly altered by the transfer from Chinese kale to cucumber, poinsettia, and tomato plants, with an impact on the first generation. Furthermore, the fecundity of *B. tabaci* was significantly reduced after being transferred from Chinese kale to cotton plants. A similar effect has also been recorded in other plant sap-sucking insects such as *Ac. pisum*, which displays lower fitness after it has been transferred from *Vicia faba* to *V. villosa*, *Medicago sativa*, and *M. truncatula* [34]. Zhang et al. [35] also reported that when *Aphis gossypii* is transferred from cotton to cowpea, pumpkin, and zucchini, the amount of *Buchnera* (the primary endosymbiont of *Ap. gossypii*) becomes unstable, and the survival rates of *Ap. gossypii* significantly decrease. Host plant shifting is, therefore, a challenge for both the directly-affected insects and their indirectly-affected endosymbionts; there are new diet conditions and defensive compounds that may require acclimation.

In the present study, the expression levels of selected amino acid biosynthesis genes of *P. aleyrodidarum* were differentially regulated when *B. tabaci* was transferred or acclimated to some host plants. This may be the result of the endosymbionts responding to physiological conditions in the insect host or the host manipulating its endosymbionts to correspond with its physiological conditions [36]. For example, the lysosomal system of *Ac. pisum* is involved with the degradation of its primary endosymbiont, *Buchnera*, and cereal weevils (*Sitophilus* spp.) also can digest and recycle their primary endosymbiont, *Sodalis pierantonius*, by cell apoptosis and autophagy activation after their exoskeleton is formed [37,38]. To overcome defensive compounds or to compensate for the different nutrient compositions in new host plants, *B. tabaci* may actively manipulate the amount and activities of its endosymbionts during host plant acclimation. The performance of *B. tabaci* may consequently be influenced.

Notably, the fecundity of *B. tabaci* was restored and the developmental time decreased after it had been reared on cotton plants for 10 generations. Also, the expression of *trpA* and *trpB* was upregulated, and that of *ilvC* and *ilvD* was downregulated after 10 generations. One possible explanation of the improved performance of *B. tabaci* is that it manipulates the expression of essential amino acid biosynthesis genes of *P. aleyrodidarum*, thus helping it to overcome the changing nutrient conditions in the phloem sap. This explanation is supported by a study revealing that none of the regulatory genes related to amino acid biosynthesis were found in *P. aleyrodidarum*, which suggests that it is *B. tabaci*, not *P. aleyrodidarum*, that can regulate the biosynthesis of amino acids [39]. Moreover, one generation may be insufficient for *B. tabaci* to respond to the different diet conditions between the phloem sap of cotton and Chinese kale, which could explain why the fecundity of first-generation whiteflies was lower than that of the original colony. Our findings suggest that the differential expression of essential amino acid biosynthesis genes of *P. aleyrodidarum* is positively associated with the performance of *B. tabaci* reared on cotton plants. Further research is warranted to investigate the mechanism of how *P. aleyrodidarum* improves the performance of *B. tabaci* on cotton plants.

The expression levels of few selected amino acid biosynthesis genes of *P. aleyrodidarum* were regulated after *B. tabaci* were transferred from Chinese kale to tomato and poinsettia for 10 generations. The differentially regulated genes varied depending on host plant and were different between males and females. Since the developmental time of *B. tabaci* increased after 10 generations, *B. tabaci* may not acclimate to poinsettia and tomato within 10 generations. The causes of differential expression between males and females are not clear. One possible reason is that females need more nutrients than males for reproduction [32]. Further research is warranted to investigate the mechanism of why the expression of the selected genes was different between males and females.

Although the expression levels of some selected amino acid biosynthesis genes of *P. aleyrodidarum* were significantly regulated after *B. tabaci* was transferred from Chinese kale to tomato, the performance of *B. tabaci* decreased. This suggests that nutritional regulation manipulated by *B. tabaci* may not be the only factor that affects whitefly performance. Other factors, such as mechanical defense and defensive compounds in plants, may also affect their performance and physiological condition. Rakha et al. [40] reported that a high density of type IV trichomes on tomato leaves reduces infestation by *B. tabaci* and increases their mortality, which suggests higher resistance toward whiteflies. In addition, we cannot rule out the possibility that the differences between the first and tenth generations could potentially be due to random fluctuations rather than actual acclimation.

Since endosymbionts have close relationships with their insect hosts in many aspects, the potential use of symbionts as tools to control insect pests and vector-borne diseases has received considerable attention in recent years [41]. Indeed, several strategies for controlling vector-borne plant diseases and insect pests of crops have been proposed to target insect endosymbionts [42]. Paratransgenesis, which modifies the symbiotic microorganisms by genetic transformation, is a conceivable approach. This has been shown, for example, by Arora et al. [43] in a study of *Pantoea agglomerans*, a symbiont of *Homalodisca vitripennis* that transmits *Xylella fastidiosa* causing Pierce’s disease in grapevines. They genetically engineered *P. agglomerans* to express antimicrobial peptides, which successfully killed *X. fastidiosa* in its insect vector [43]. Therefore, a greater understanding of the interactions among *B. tabaci*, its endosymbionts, and the host plant may lead to novel strategies in the control of *B. tabaci*.

## 5. Conclusions

The present results demonstrate that host shifting from Chinese kale to cotton plants leads to a decrease in the performance of *B. tabaci* in the first generation, which is restored by the tenth generation. Furthermore, after *B. tabaci* has acclimated to the cotton plants, the expression levels of essential amino acid biosynthesis genes of its primary endosymbiont, *P. aleyrodidarum*, are differentially regulated. The present study suggests that *P. aleyrodidarum* may help *B. tabaci* improve its performance and acclimate to new host plants and that *P. aleyrodidarum* has a close nutritional relationship with its host during host plant acclimation.

## Figures and Tables

**Table 1:** Primers used to determine the expression levels of essential amino acid biosynthesis genes of *P. aleyrodidarum*.

Gene	ID ^1^	Sequence (5′ to 3′)	Amplicon
*trpA*	Por0185	trpA-F: ACCACCAGAACACGCTCAAGAA trpA-R: CTTCCTCCCGTAACTCCATTTATTG	165 bp
*trpB*	Por0186	trpB-F: CGGGTCTAACGCAATGGGAT trpB-R: ACTCCAGGCACACCTCCATTTAA	135 bp
*leuB*	Por0099	leuB-F: TGGTATAGGGCCCGAAGTGATC leuB-R: AGCGAGATGCTTTTGCTGCTT	170 bp
*leuC*	Por0263	leuC-F: CAGATGGAGGCACAGGATATGC leuC-R: CCAACTTTTGCTCCTGCTTCAA	115 bp
*ilvC*	Por0140	ilvC-F: TGTGGTGGCGTATCAGCATTAA ilvC-R: TCACCCAAACCCCCTTCATATA	140 bp
*ilvD*	Por0090	ilvD-F: AGGCAATGGGACGTTGTTATCA ilvD-R: CCACCCATTGCTATATCCATTATCAT	180 bp
*gyrA*	Por0266	gyrA-F: TATGGCAACGAATGTACCTCCTC gyrA-R: ACAATCCCAGCAGTCCCATAAAT	159 bp

^1^ Gene ID at whitefly genome database (http://www.whiteflygenomics.org).
